# Introduction to ISBGMO12: biosafety research past, present and future

**DOI:** 10.1007/s11248-014-9794-z

**Published:** 2014-05-14

**Authors:** Alan Raybould, Hector Quemada, Jörg Romeis

**Affiliations:** 1 Syngenta, Jealott’s Hill International Research Centre, Bracknell, Berkshire, RG42 6EY UK; 2 Donald Danforth Plant Science Center, St. Louis, MO 63132 USA; 3 Agroscope, Institute for Sustainability Sciences ISS, Reckenholzstr. 191, 8104 Zurich, Switzerland

The International Symposium on Biosafety of Genetically Modified Organisms (ISBGMO) is a biennial international meeting organised by the International Society for Biosafety Research (ISBR; www.isbr.info/). ISBR promotes research and application of science in the fields of agricultural and environmental biotechnology and risk analysis. In particular, ISBR encourages research that supports the safe and effective use of biotechnology in agriculture, food production, and public health, and assists the development of the relevant policy and regulation.

The first ISBGMO was held in Kiawah Island, North Carolina, USA in November 1990. Since then, many countries have hosted the meeting: Germany (twice), USA (California), Japan, Canada, China, France, South Korea, New Zealand and Argentina. The 12th ISBGMO was held in St Louis, Missouri, USA from the 16th to the 20th of September 2012, and was attended by about 500 delegates from 47 countries.

This special section of *Transgenic Research* features thirteen papers developed from lectures and workshops at the meeting. Taken together, the papers indicate that, for first generation genetically modified (GM) crops at least, biosafety research is increasingly addressing questions about the sustainable deployment of the crops in agricultural systems, and is focussing less on the basic characteristics of GM crops as a class. Making regulatory risk assessment efficient and effective, and realising the economic, environmental and social opportunities presented by commercialised GM crops, now seems more important than, say, further basic research on unintended effects of transformation or gene flow from GM crops to wild species. This is a hugely significant development.

While ISBR and ISBGMOs focus on science, the role of policy in framing research questions and ensuring effective application of new knowledge is increasingly recognised. In his keynote address, Raven (2013) placed GM crops in the context of fulfilling agricultural policy. If our policy is to increase food production to meet increasing need, then GM crops ought not to be singled out for “burdensome” regulatory treatment: “it is no longer acceptable to delay the use of any strategy that is safe and will help us achieve the ability to feed the world’s people.”

A source of onerous regulatory treatment was concern that that GM crops might “escape”. At the time of the first ISBGMO in 1990, scientists were thinking about the ecological consequences of transgenes not being contained by agricultural management. One concern was that genetically modification of crops would create more serious weeds of agriculture or plants more invasive of non-agricultural habitats. These problems may occur if the introduced trait provides tolerance or resistance to a factor that was controlling the persistence or spread of the crop (Keeler 1989)—a process called ecological release (Schmitt and Linder 1994). A more speculative mechanism for ecological release was that transformation would lead to unintended effects, perhaps through disruption of native genes, pleiotropy or epistasis (Regal 1994).

Discussions about ecological release of GM crops and unintended changes caused by transformation focussed on technical questions, such as the mechanisms by which unintended effects may arise, and the potential changes in the population dynamics of feral GM crops or hybrids. Often such discussions missed a crucial element of risk assessment: what changes ought to be regarded as harmful? Without agreed definitions of harm from using a GM crop, biosafety research often confuses rather than clarifies risk assessments. Without harm as a context for evaluating the significance of changes, regulatory decision-making becomes “burdensome” as it tries to catalogue all possible changes that might arise from using a GM crop, rather than the likelihood and seriousness of harmful effects.

This question of how to define harm was central to several papers presented at ISBGMO12. Devos et al. (2013) point out that disputes about the risks—and opportunities—from using GM crops may result from differences in values. In such circumstances, clarification of policy objectives is vital. Without such clarity, additional scientific research may not help people to reach agreement, and indeed may make disagreements worse. Gray (2013) reinforces this point with examples from applied ecology. He notes that misunderstandings between scientists and policymakers are not unique to GM crops, although the debate about the use of GM crops may be uniquely intense.

The benefits of clarifying policy objectives are demonstrated in the papers by Andrade et al. (2014) and Nang’ayo et al. (2014), writing about Brazil and Africa respectively. In Brazil, there is clarity: post-market monitoring concentrates on detecting harmful effects from cultivating a GM crop, not on comparing the agro-ecosystems where the GM crop is cultivated with a baseline of conventional (i.e., non-GM) crop cultivation. The advantages of the system are flexibility, cost-effectiveness and proportionality. In Africa, on the other hand, there is ambiguity. GM crops provide opportunities to fulfil policy objectives of improving the lives of smallholders through higher yields and better quality of crops, while simultaneously reducing unsuitable use of pesticides and fertilisers. Nevertheless, many countries in Africa also have policies that result in regulations that constrain or prevent research and development of GM crops tailored to local conditions and needs.

A weight of evidence from field studies of GM crops in uncultivated land (e.g., Crawley et al. 1993; 2001), phenotypic comparisons of GM crops with non-GM near isolines (e.g., Horak et al. 2007), and molecular analysis of genetic changes induced by transformation and other breeding methods (e.g., Ricroch et al. 2011) suggests that GM crops are no more likely to be serious weeds than are other new crop varieties. Nevertheless, new data are often required to complete regulatory assessments of the weediness and invasiveness of GM crops (e.g., Raybould et al. 2012). Three papers from ISBGMO12 may help to reduce or eliminate these requirements.

First, Garcia-Alonso and Raybould (2013) describe how general concerns about the effects of weedy or invasive GM crops may be translated into operational definitions of the environmental entities that are to be protected. Secondly, Keese et al. (2013) describe risk assessments for importing plants new to Australia, and how these established methods may be applied to the risks posed by cultivating GM crops. Finally, Roberts et al. (2013) point out that assessment of the risks of weedy or invasive crops too often focuses on exhaustive characterization of potential hazards, when an estimate of exposure—the amount of gene flow from the GM crop through pollen or seed—would be sufficient to conclude with high confidence that the risks are minimal. By explicitly stating objects of concern, and putting the risks from GM crops in context with those from other plants, these papers will help risk assessors to judge whether data requirements for weediness assessments are proportionate.

In addition to gene flow, weediness and invasiveness, a recurrent theme of ISBGMOs has been the potential effects of *Bt* crops on non-target organisms (NTOs). Between 2006 and 2012 sound theoretical and practical frameworks for risk assessment and testing methods covering *Bt* crops and NTOs were developed and published (Garcia-Alonso et al. 2006; Romeis et al. 2008, 2011, 2012). As Burns and Raybould (2013) show, these methods are becoming routine for regulatory risk assessments for GM insect-resistance traits in the USA.

NTO risk assessments often make conservative assumptions about which organisms will be exposed to *Bt* proteins. Hence in many cases, studies of the hazard of proteins to groups of NTOs may be unnecessary because those groups are unlikely to be exposed. Romeis et al. (2014) present a database that contains bio-ecological information on arthropods found in relevant agro-ecosystems in Europe. This information could help focus ecological risk assessments by identifying NTOs that are ecologically important and that are likely to be exposed. Better knowledge of the ecology the non-target fauna in crops may reduce the amount of hazard testing needed for ERAs of insect-resistant GM crops.

We often hear that risk assessment for GM crops must be case-by-case. With a huge variety of potential traits, environments and policies, a risk assessment for a particular GM crop in a particular country cannot cover all uses of all GM crops. Nevertheless, case-by-case does not mean that each risk assessment must start from scratch; each risk assessment may contain data and analysis that are useful for subsequent risk assessments. Two papers presented at the conference describe different aspects of this topic.

Kearns et al. (2013) describe the work of the Organisation for Economic Co-operation and Development (OECD) in producing consensus documents for risk assessment of GM crops. These documents compile information on the biology of crops and traits that are agreed to be relevant to comparative risk assessment. The OECD documents mean that regulatory authorities need not always start from scratch when faced with a crop or trait new to their country.

Garcia-Alonso et al. (2014) tackle the question of whether field trial data must be produced in the country to which the risk assessment applies. They propose a conceptual framework, based on agro-climatic zones, for determining whether data produced in one country are relevant to other countries. If accepted, the framework will allow researchers to design trials that produce data for use in many countries. The framework would reduce the time and expense of producing regulatory data without compromising the rigour of risk assessments produced from those data.

Finally, technology development and application is continuous. While biosafety research on herbicide-tolerant and insect-resistant GM crops may have moved from fundamental questions about their properties to applied questions about effective risk assessment and regulation, we may need to return to basic research for crops developed from new technology. Furthermore, as Hokanson et al. (2013) show, not all future products of agricultural biotechnology will be crops. Nevertheless, experience from first-generation GM crops teaches us that regulation ought to be designed to deliver clear policy objectives about real products. This experience will also help us to identify data that are essential for assessing the risks from future products.
